# Out of the cave: Rewilding deep time at the Venice Biennale

**DOI:** 10.1016/j.isci.2025.113392

**Published:** 2025-09-02

**Authors:** Gabriela Amorós, José S. Carrión, Federica Crivellaro, Ana B. Marín-Arroyo

**Affiliations:** 1 Departamento de Biología Vegetal, Universidad de Murcia, 30100 Murcia, Spain; 2 Via Trastevere 94, 00153 Roma, Italy; 3 Grupo EVOADAPTA, Departamento de Ciencias Históricas, Universidad de Cantabria, 39005 Santander, Spain

## Abstract

*Out of the Cave*, part of the 2025 Venice Architecture Biennale, reimagines prehistoric human life through scientifically grounded paleoart. This visual installation bridges deep-time knowledge with contemporary ecological and ethical issues. By integrating paleoecological data, biodiversity representation, and dietary insight, the project invites reflection on sustainability and modernity. This Backstory outlines the scientific and conceptual foundations of the artwork, offering a model for transdisciplinary practice at the intersection of biology, anthropology, and spatial design.

**Figure:**
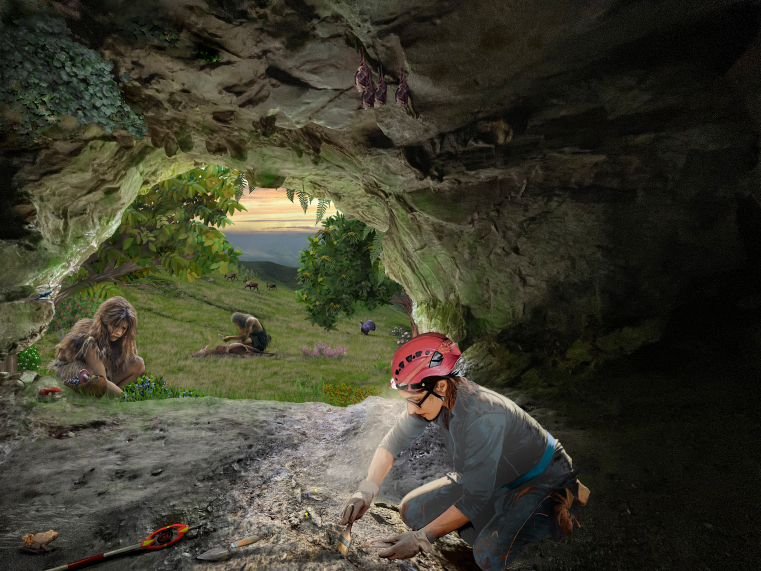
Above image: Principal element of the exhibition panel “*Out of the Cave*” at the 2025 Venice Biennale: a paleoartistic reconstruction based on paleoecological data from the Late Pleistocene of the Cantabrian region of Spain, onto which elements of prehistory, current and past biodiversity, and symbolic motifs are incorporated to convey a conservationist message. Copyright: G. Amorós.

**Figure:**
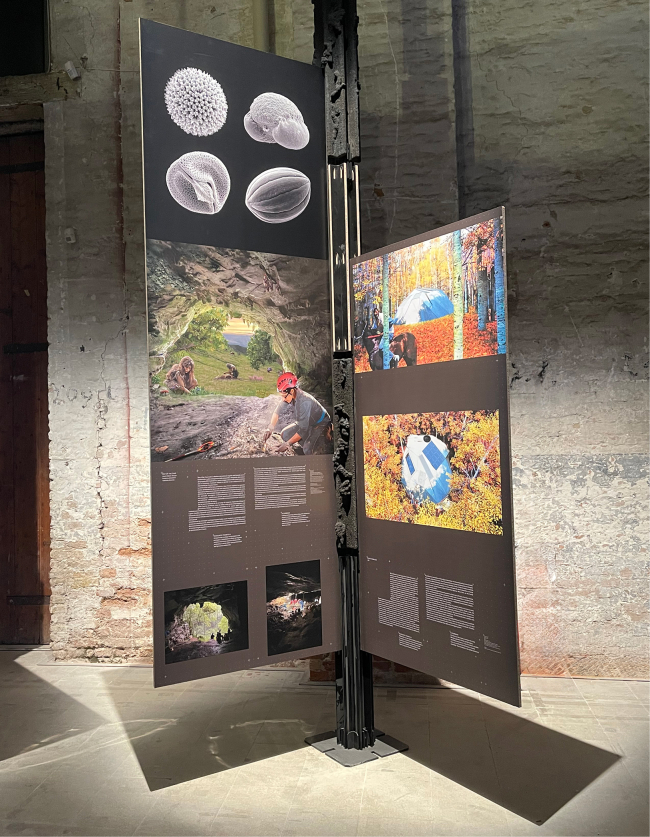
*Out of the Cave* in its designated section at the Biennale. At the top, scanning electron microscope images of pollen grains from plants that existed in the region’s prehistoric past. In the center, a symbolic paleoartistic reconstruction. Below, photographs of ongoing excavations in the Cantabrian Paleolithic.

“Art has the potential to support a redirection of the conservation agenda, combining scientific awareness with imaginative engagement.”

“Promoting integrative and transdisciplinary approaches within both research and education is not only timely—it is essential.”

## Main text

### Introduction

We present *Out of the Cave*, an installation selected for inclusion in the 19th International Architecture Exhibition of La Biennale di Venezia—*Intelligens. Natural. Artificial. Collective*. *https://www.labiennale.org/en/news/biennale-architettura-2025-intelligens-natural-artificial-collective*—open to the public from May 10 to November 23, 2025. In the face of accelerating climate disruption, the 2025 Biennale reimagines architecture as a transdisciplinary act of adaptation. The very title, *Intelligens*, merges “intelligence” and *gens* (Latin for “people”), signaling a paradigm shift that integrates natural, artificial, and collective intelligences to rethink how we design for a world in flux. Within this framework, architecture becomes more than shelter—it becomes a conscious way of inhabiting the planet with ecological awareness and ethical responsibility.

*Out of the Cave* is conceived as a predominantly visual piece that communicates through scientific imagery, with minimal reliance on text (Image 1). The project emerged from collaboration between a paleoartist with a background in biology (Gabriela Amorós), a paleobiologist with a passion for art (José S. Carrión), an evolutionary anthropologist curating scientifically grounded exhibitions (Federica Crivellaro), and a prehistorian focused on climate change and paleodiet (Ana B. Marín-Arroyo) (Image 1). We propose a novel and timely perspective by reframing knowledge about prehistoric human life within the context of contemporary and future architectural thinking. This ambition resonates with Edward O. Wilson’s^1^ insight that “history makes little sense without prehistory, and prehistory makes little sense without biology.”

### Art and science

#### Bridging temporalities through inclusive paleoart

*Out of the Cave* invites viewers to shift their perspective from the microscopic to the ecosystemic, weaving together photographs of recent archaeological excavations and pollen grains with a layered temporal scene that merges scientific reconstruction and symbolic reflection (Image 2). Part of this artwork can be situated within what Amorós (2024)^2^ describes as “paleoart”—a practice that integrates paleoecological data to “bring to life” scenes from the distant past (Image 1). In our case, since paleoart has traditionally been zoocentric,^2^ privileging large fauna, we place special emphasis on plant life^3^ and include other animal groups such as birds, bats, amphibians, and insects (supplementary files). Created using professional digital illustration tools, including Adobe Photoshop and a high-resolution graphic tablet, the piece depicts two women—separated by tens of thousands of years—inhabiting the same physical space, yet belonging to vastly different temporalities (Image 1). Within the artificially lit interior, a present-day archaeologist conducts an excavation, while near the cave’s entrance, a prehistoric woman gathers berries, nuts, and mushrooms. Outside, a Paleolithic man butchers a hunted deer, with a herd of reindeer visible in the distance. This layered tableau collapses temporal boundaries, prompting critical reflection on both ancient lifeways and the contemporary practices through which we access, reconstruct, and narrate them.

Much of this reconstruction is based on the study and interpretation of fossil pollen and macroremains retrieved from archaeological sediments and adjacent depositional basins such as lakes and peat bogs.^4^ The scene illustrates the Cantabrian region during a substantial portion of the Late Pleistocene and likely evokes a landscape from around 35,000 years ago^5^ (Image 1). During cold phases of the Pleistocene, the region experienced alternating episodes of semi-forested conditions (interstadials) and more open environments dominated by herbs and shrubs (stadials), with a prevalence of grasses (*Poaceae*) and heathers (*Erica* and *Calluna*), along with sedges (*Cyperaceae*), plantains (*Plantago*), horsetails (*Equisetum*), and ferns—here, notably *Polypodium*^4^ (Figure S1). In the background of the artwork, suggesting the presence of sublittoral and intramontane tree refugia, small stands of silver fir (*Abies alba*) are included. Near the cave, several woody species bearing edible fruits—such as walnut (*Juglans regia*), sweet chestnut (*Castanea sativa*), and rowan (*Sorbus aucuparia*)—appear alongside other potentially culinary resources, including *Vaccinium* and *Ribes* species (supplementary files).^4^

#### Seeing biodiversity differently

The way we choose to depict biodiversity reflects not only aesthetic preferences but also ethical beliefs. In *Out of the Cave*, this act of *seeing*—of rendering visible what is often overlooked—becomes a means of rethinking our relationship with nonhuman life. While it is often said that the devil is in the details, here those details—both taxonomic and ecological—offer insight rather than mischief. Art has the potential to support a redirection of the conservation agenda, combining scientific awareness with imaginative engagement. Several of the species depicted in the scene function as bioindicators of environmental quality or as remnants of broader historical ranges (Figure S2). For instance, the reindeer (*Rangifer tarandus*), adapted to Pleistocene cold climates, once roamed across much wider territories than it does today.^6^ *Rosalia alpina* (shown on the rocky ledge next to the gathering woman), with its striking bluish-gray coloration and black markings, is a saproxylic beetle typical of mature beech and oak forests, now declining due to intensive logging.^7^ The midwife toad (*Alytes obstetricans*), visible in the lower left corner, is widely regarded as a bioindicator of ecological integrity.^8^ Thalloid liverworts, growing on the ceiling of the cave, are highly sensitive to air pollution. *Tetrao urogallus* (capercaillie), found beneath the chestnut tree to the right, is a critically endangered species of structured boreal-alpine forests and has become emblematic for conservation biologists. The lesser horseshoe bat (*Rhinolophus ferrumequinum*), a flagship species, is now endangered in many regions due to habitat degradation and pesticide use.^9^ Depicting these species without placing them in the visual foreground is part of a pedagogical strategy: in nature, countless interconnections among plants, animals, fungi, and microorganisms go largely unnoticed (Image 1). In so-called “modern societies,” species and ecosystems are often reduced to providers of services, stripped of their inherent value and beauty. Yet each species is a unique living structure—shaped by deep time, forged through complex evolutionary trajectories, and, against all odds, now coexisting with us in the intricate web of life.

#### Food for thought and health

Food is far more than a source of calories—it shapes social bonds, expresses cultural identities, drives economies, supports human health, and inspires innovation. *Out of the Cave* offers a lens into the dietary practices of our prehistoric ancestors, presenting both literal and metaphorical “food for thought” as we confront an ecologically uncertain future. Beyond the omnivorous idiosyncrasy of our species—evidenced here by the consumption of reindeer, deer, berries, nuts, and mushrooms—the archaeological record reveals a remarkable degree of dietary versatility in early *Homo sapiens.*^10^

Early humans also lived in ecosystems teeming with pathogens,^11^ where survival depended not only on shelter and tools but also on a deep knowledge, often acquired through cumulative cultural learning and empirical observation, of both nutritional and medicinal resources. In such infection-prone environments, a diet rich in bioactive compounds likely played a critical role in supporting immune function and overall resilience.^11^ *Vaccinium myrtillus* (bilberry) is one such wild food, rich in flavonoids—particularly anthocyanins—with well-documented antioxidant, anti-inflammatory, and vasoprotective properties, offering potential benefits across a wide range of infectious diseases.^12^ Similarly, edible mushrooms gathered in the wild provide not only nutritional value but also bioactive polysaccharides such as β-glucans, which are known to enhance innate immunity and support host defense mechanisms^13^ (Figure S1).

The presence of *Lepista nuda* (in the basket), in contrast to *Amanita muscaria* (at the cave entrance, near the woman), also reflects a form of prehistoric ecological knowledge (Figure S1). While *Lepista nuda* is edible, it requires accurate identification to avoid confusion with toxic look-alikes. *Amanita muscaria*, on the other hand—though toxic—may have been used ceremonially; its cap contains psychoactive compounds and has been employed in ritual contexts by some contemporary cultures when properly dried and detoxified.^14^

#### From myth to meaning

Plato’s famous Allegory of the Cave, told in *The Republic*, describes prisoners who have spent their entire lives chained inside a cavern, forced to gaze only at a wall where shadows are projected—cast by objects passing before a fire behind them. For these prisoners, the shadows constitute the only reality they know. The allegory illustrates the contrast between the world of appearances, perceived through the senses, and the true world, accessible only through reason and knowledge.

*Out of the Cave* invites visitors into a dimly lit space that evokes the habitat of early *Homo sapiens* (Image 1). On the surface, one enters a scientifically informed reconstruction of a remote past; yet the experience also prompts a critical reflection on the present. What appears at first as a play of evidence-based projections—shadows of the past—gradually confronts us with an uncomfortable truth: modern societies have strayed from the sustainable lifeways that once ensured human survival. In this light, Plato’s allegory acquires renewed relevance. Just as the prisoners mistook shadows for reality, today’s world often mistakes exploitation for development. The challenge, then, is not only to look at the past but also to see through the shadows of the present.

### Challenges and opportunities

#### Reflections on art and science

The expressive and imaginative power of paleoart remains underutilized in scientific communication. Yet this power stems from shared cognitive roots between scientific reasoning and artistic creation. Despite this, science is often perceived as distant from creativity, a perception reinforced by rigid academic structures that separate knowledge into artificial compartments. This division limits the potential for innovation and obstructs the kind of holistic thinking needed to address the complex challenges of our time. Promoting integrative and transdisciplinary approaches within both research and education is not only timely—it is essential. *Out of the Cave* emerges from this conviction that the boundaries between disciplines must be softened and that new ways of seeing, knowing, and acting can arise when science engages with the visual and symbolic languages of art.

## Acknowledgments

The research presented in the 19^th^ International Architecture Exhibition of La Biennale di Venezia, curated by Carlo Ratti, has been primarily funded by the European Research Council under the European Union’s Horizon 2020 Research and Innovation Programme (grant no. 818299; SUBSILIENCE project; https://www.subsilience.eu). The paleoart presented is inspired, in part, by the Aitzbitarte III cave. Since 2022, Aitzbitarte III excavations have been directed by A.B.M.-A. and permitted by the Diputación Foral de Guipuzkoa (Dpt Cultura). The PALARQ foundation funded some analytical work in Aitzbitarte III under the 2024 funding programme. J.S.C. and G.A. acknowledge funding by Agencia Estatal de Investigación project HOMEDSCAPE PID2022-136832NB-100 and Fundación Séneca Región de Murcia, project 22525/PI/24.
