# Supplementary material for: Out of the cave: Rewilding deep time at the Venice Biennale
Source: iScience. 2025 Sep 2;28(9):113392. doi: 10.1016/j.isci.2025.113392 (PMC12424228; doi:10.1016/j.isci.2025.113392)

iScience, Volume *28*

Supplemental information

Out of the cave: Rewilding deep time at the Venice Biennale

Gabriela Amorós, José S. Carrión, Federica Crivellaro, and Ana B. Marín-Arroyo

Figure 1

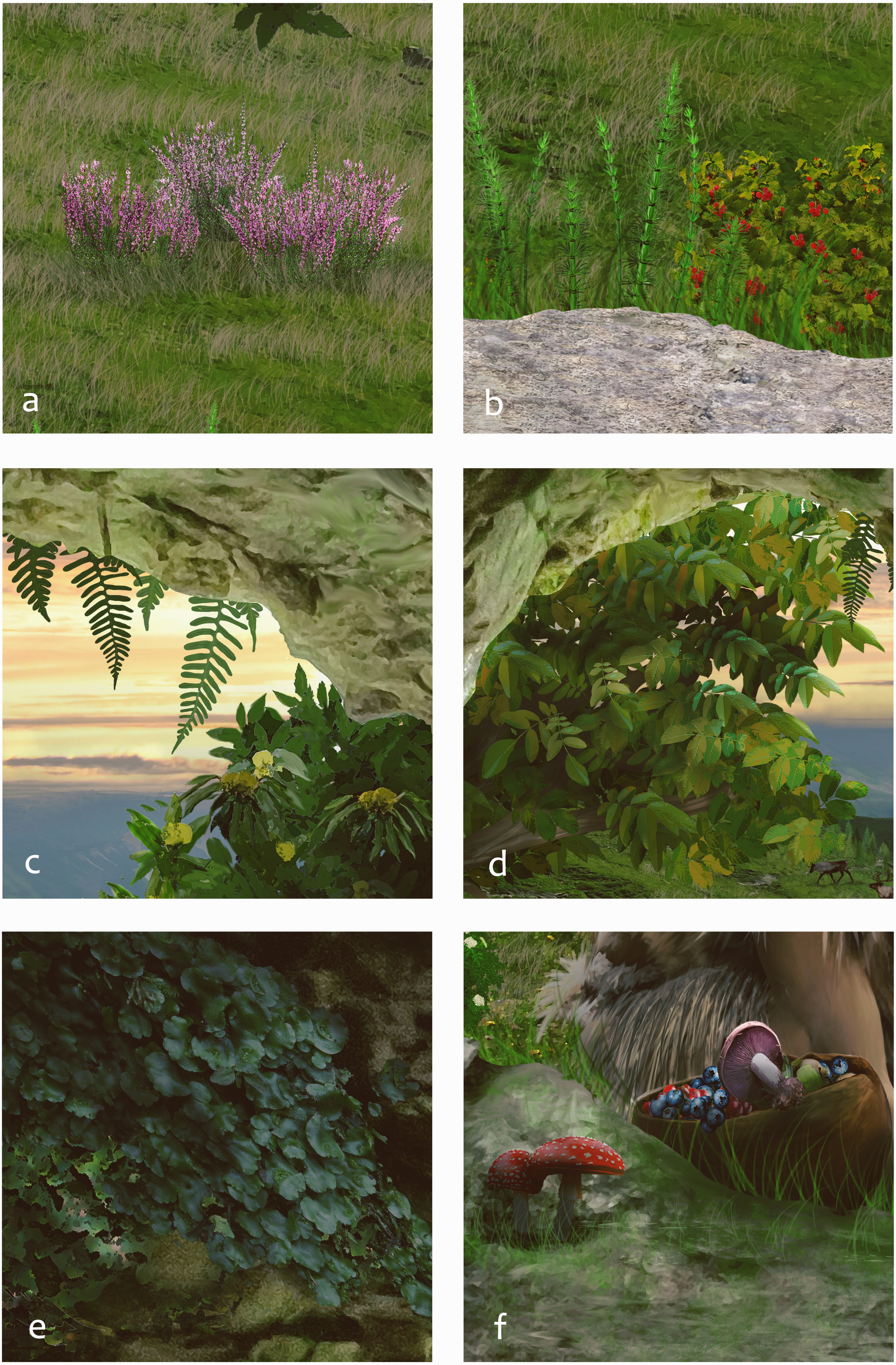

Figure 2

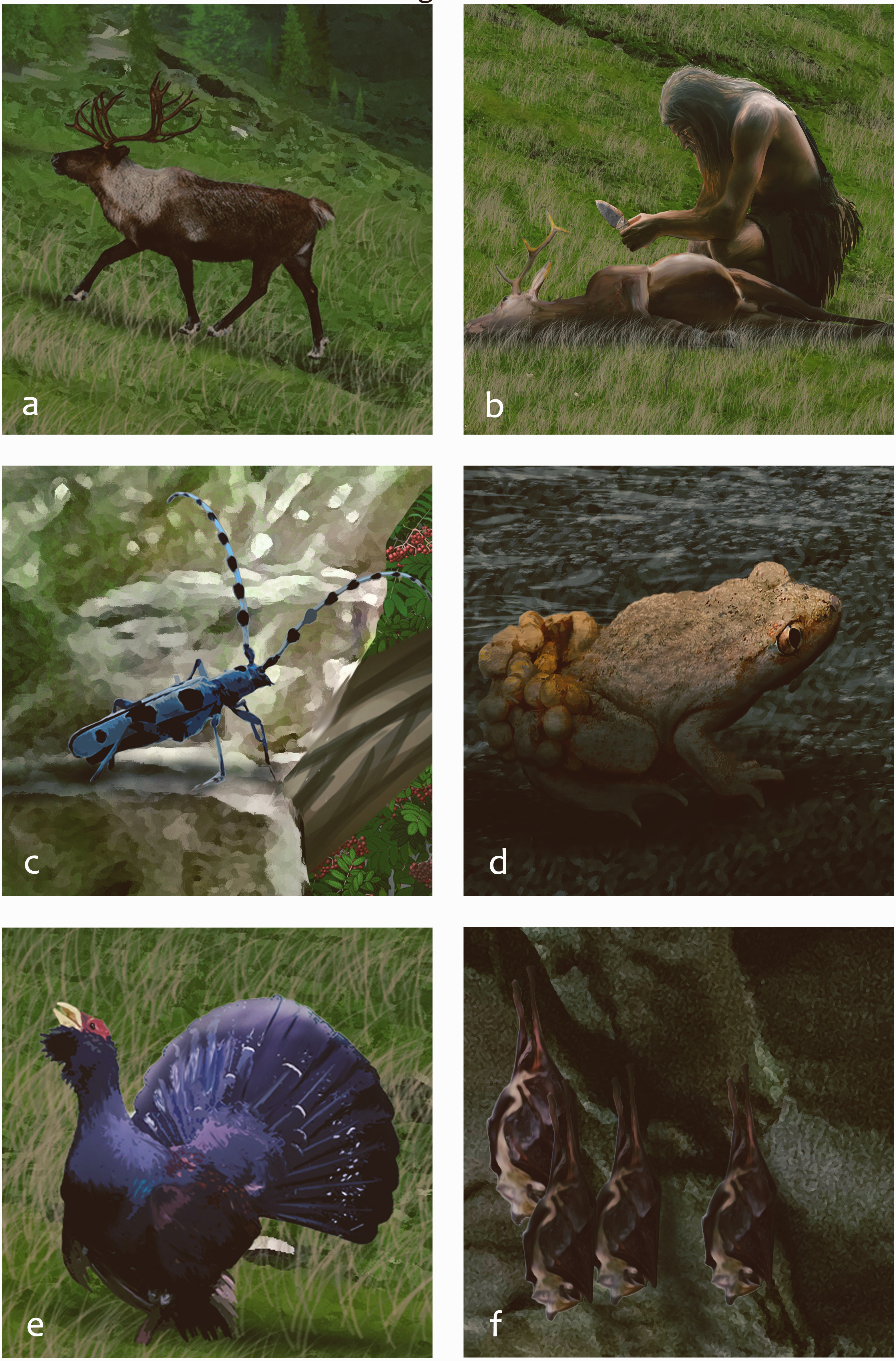

Supplement: Document S1. Figures S1 and S2 [file mmc1.pdf]
